# Calcium-Oxidant Signaling Network Regulates AMP-activated Protein Kinase (AMPK) Activation upon Matrix Deprivation*

**DOI:** 10.1074/jbc.M116.731257

**Published:** 2016-05-11

**Authors:** Ananthalakshmy Sundararaman, Usha Amirtham, Annapoorni Rangarajan

**Affiliations:** From the ‡ Department of Molecular Reproduction, Development and Genetics, Indian Institute of Science, Bangalore-560012 and; the § Department of Pathology, Kidwai Memorial Institute of Oncology, Bangalore-560030, India

**Keywords:** AMP-activated kinase (AMPK), anoikis, calcium, cancer biology, reactive oxygen species (ROS), matrix deprivation

## Abstract

The AMP-activated protein kinase (AMPK) has recently been implicated in anoikis resistance. However, the molecular mechanisms that activate AMPK upon matrix detachment remain unexplored. In this study, we show that AMPK activation is a rapid and sustained phenomenon upon matrix deprivation, whereas re-attachment to the matrix leads to its dephosphorylation and inactivation. Because matrix detachment leads to loss of integrin signaling, we investigated whether integrin signaling negatively regulates AMPK activation. However, modulation of focal adhesion kinase or Src, the major downstream components of integrin signaling, failed to cause a corresponding change in AMPK signaling. Further investigations revealed that the upstream AMPK kinases liver kinase B1 (LKB1) and Ca^2+^/calmodulin-dependent protein kinase kinase β (CaMKKβ) contribute to AMPK activation upon detachment. In LKB1-deficient cells, we found AMPK activation to be predominantly dependent on CaMKKβ. We observed no change in ATP levels under detached conditions at early time points suggesting that rapid AMPK activation upon detachment was not triggered by energy stress. We demonstrate that matrix deprivation leads to a spike in intracellular calcium as well as oxidant signaling, and both these intracellular messengers contribute to rapid AMPK activation upon detachment. We further show that endoplasmic reticulum calcium release-induced store-operated calcium entry contributes to intracellular calcium increase, leading to reactive oxygen species production, and AMPK activation. We additionally show that the LKB1/CaMKK-AMPK axis and intracellular calcium levels play a critical role in anchorage-independent cancer sphere formation. Thus, the Ca^2+^/reactive oxygen species-triggered LKB1/CaMKK-AMPK signaling cascade may provide a quick, adaptable switch to promote survival of metastasizing cancer cells.

## Introduction

Epithelial cells are known to be dependent on interaction with specific extracellular matrix components for their survival (1). Lack of matrix attachment triggers a form of caspase-mediated apoptotic cell death called anoikis (2). Anoikis serves as a formidable barrier to metastasis. Cancer cells overcome anoikis through various genetic and epigenetic changes that sustain the survival pathways even under detached conditions (3). During metastasis, cancer cells leaving the primary tumor site are deprived of matrix attachment in circulation, while they may be exposed to foreign matrix components at the secondary site that may not be conducive for attachment (4). Besides, during the initial stages, glandular epithelial cancer cells that are shed into the lumen of the duct or lobule resulting in luminal filling are also matrix-deprived. Therefore, understanding the molecular mechanisms that promote cancer cell survival under matrix-deprived conditions becomes relevant. Recent studies have thrown light on the metabolic alterations in extracellular matrix (ECM)^3^-deprived cells. Anchorage deprivation has been linked to reduced glucose uptake and increased ROS levels (5). Recent work from our laboratory has shown that matrix-deprived cells activate a central metabolic regulator AMP-activated protein kinase (AMPK), which contributes to anoikis resistance through phosphorylation of PEA15 (6). Moreover, AMPK-mediated suppression of mTORC1 (7) and maintenance of NADPH homeostasis by inhibition of ACC (8) contribute to anoikis resistance. However, the molecular mechanisms that lead to AMPK activation in the context of loss of matrix attachment remain unexplored.

Cell attachment to extracellular matrix is mediated by a family of adhesion proteins called integrins. Integrins are implicated in tumor proliferation, survival, invasion, and migration. These transmembrane proteins are heterodimers composed of α and β chains. When attached to ECM proteins like collagen, elastin, laminin, or fibronectin, integrins cluster on the membrane and recruit various signaling and adaptor proteins to form focal adhesions. The formation of focal adhesions lead to activation of focal adhesion kinases (FAK), Src family kinases, and p130^CRK^ associated substrate (p130^CAS^) (9). Integrin-mediated signaling plays a central role in suppressing apoptosis in adherent cells; loss of integrin downstream signaling through FAK and Src has been implicated as a major cause of anoikis (10). Although matrix deprivation leads to loss of integrin signaling on the one hand and AMPK activation on the other hand, the cross-talk between these two signaling pathways has not been explored.

AMPK has been described as the “fuel gauge of the cell.” AMPK is regulated by phosphorylation at a key threonine residue at position 172 in the activation loop of the α subunit (11). Lowered cellular ATP levels and an increase in AMP are known to activate AMPK allosterically and prevent its dephosphorylation at Thr-172. Two upstream kinases, LKB1 and CaMKKβ, are known to majorly phosphorylate AMPK at Thr-172 and activate it. LKB1 is known to mediate AMPK activation under conditions of energy stress. While LKB1 is known to mediate AMPK activation under conditions of energy stress, CaMKKβ activates AMPK in an AMP-independent manner, in response to an increase in cytosolic calcium (12).

Activation of AMPK can also occur in response to oxidative stress (13). However, the mechanisms that activate AMPK in this context remain unclear. Oxidative stress can induce changes in the AMP/ATP ratio, leading to AMPK activation (14). Conversely, AMP-independent mechanisms like store-operated calcium entry (SOCE), in response to oxidative stress, are also known to activate AMPK (15). ROS and calcium are thus known to regulate AMPK in a context-dependent manner.

In this study, we demonstrate that AMPK is activated in a rapid and sustained manner upon matrix deprivation. We find evidence to support a role for both upstream kinases LKB1 and CaMKKβ in the activation of AMPK upon matrix deprivation, whereas LKB1-deficient cells depend predominantly on CaMKKβ. We further show an increase in calcium and ROS levels under detached conditions, and these signaling molecules contribute to rapid AMPK activation in this context. We further demonstrate for the first time a calcium surge-dependent increase in ROS levels upon detachment. We also find that the AMPK signaling pathway is active in breast cancer patients, and there is a positive correlation between LKB1 and pACC levels suggesting AMPK activation through LKB1. Thus, this study uncovers a novel mechanism for AMPK activation upon matrix deprivation, which majorly depends on the intracellular calcium and oxidant signaling.

## Results

### 

#### 

##### Rapid and Sustained Activation of AMPK upon Detachment

**FIGURE 1.**
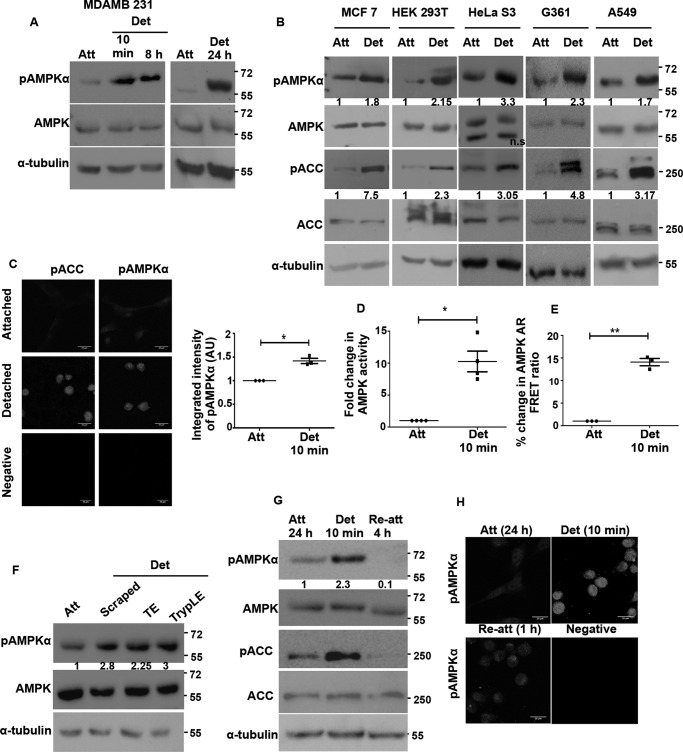
**Rapid and sustained activation of AMPK under detachment-induced stress.** *A,* MDA-MB 231 cells were cultured under adherent conditions or detached by trypsinization and subjected to suspension for the various indicated times prior to harvesting. The levels of AMPK phosphorylated at threonine 172 (pAMPKα) and total AMPK were determined by Western blotting (*n* = 3). α-Tubulin is used as loading control in all blots. Molecular size markers are depicted on all blots on the *right margin. B,* multiple cancer cell lines were cultured under attached (*Att*), or detached (*Det* 10 min) conditions. The levels of pAMPKα, pACC, total AMPK, and ACC were determined by Western blotting. The *numbers* indicate relative pAMPKα/AMPK ratio and pACC/ACC ratio (*n* = 4). In all subsequent experiments, unless otherwise mentioned, cells were detached for 10 min. *ns,* non-significant. *C,* immunocytochemistry was performed on MDA-MB 231 cells cultured under attached and detached conditions for pAMPKα (Thr-172) and pACC (Ser-79). The representative pictures are maximum intensity projections of confocal *Z* stack images. *Scale bar,* 20 μm. Total integrated pixel intensity per cell was quantified for 30 cells in each experiment. Scatterplot depicts fold change in integrated intensity of pAMPKα with each dot constituting one biological experiment normalized to the corresponding attached value (*n* = 3); *, *p* < 0.05. *Error bars* represent ± S.E. *AU,* arbitrary units. *D,* G361 cells were cultured under attached and detached (10 min) conditions. AMPK was immunoprecipitated from the lysates, and AMPK activity was measured by the incorporation of radioactive phosphate on AMARA peptide. *Graph* depicts fold change in AMPK activity (*n* = 4); **, *p* < 0.01. *Error bars* represent ± S.E. *E,* HEK 293T cells stably expressing AMPK activity reporter FRET construct (*AMPK-AR*) were cultured under attached and detached (10 min) conditions, and the FRET ratio (CY/CC) between acceptor emission (YFP emission) on donor excitation (cyan excitation) abbreviated as CY to donor emission (cyan emission) on donor excitation (cyan excitation) abbreviated as CC was measured in a spectrofluorometer. Scatterplot depicts FRET ratios expressed as a percentage of attached (*n* = 3 biological samples each with three technical replicates); *, *p* < 0.05. *F,* HEK 293T cells were detached using different modes as indicated. Cells were either scraped gently into media or detached with trypsin-EDTA (*TE*) or TrypLE and resuspended in media for 10 min. Cells were then harvested; pAMPKα and pACC levels were measured by Western blotting (*n* = 4). The *numbers* indicate relative pAMPKα/AMPK ratio. *G,* MDA-MB 231 cells were trypsinized and kept detached for 10 min or allowed to reattach to dishes immediately after trypsinization, for a period of 4 h, and the levels of pAMPKα and AMPK were determined by Western blotting (*n* = 3). *Numbers* indicate relative pAMPKα/AMPK ratio. *H,* MDA-MB 231 cells cultured under detached conditions for 10 min were compared with those that were trypsinized and allowed to attach in regular tissue culture dishes for 1 and 24 h, respectively, by immunocytochemistry for pAMPKα. The representative pictures are maximum intensity projections of the confocal *Z* stack images (*n* = 3).

Recent studies (7, 8, 25), including work from our laboratory (6), have identified AMPK activation upon matrix deprivation and its role in anoikis. To begin to understand the molecular mechanisms that lead to AMPK activation on matrix deprivation, we first investigated the status of AMPK signaling in MDA-MB 231 breast cancer cells upon detachment by culturing cells for various time periods in suspension. We performed immunoblotting to study the levels of phosphorylation of AMPKα, using an AMPKα Thr-172-phosphospecific antibody, as a measure of AMPK activation (12). We found that MDA-MB 231 cells showed elevated pAMPKα Thr-172 levels as early as 10 min following detachment, and this was sustained even at 24 h of detachment (Fig. 1*A*). Previously we and others have shown elevated pAMPK levels at 24 h following detachment as well as in cancer spheres generated in 7 days of suspension culture (5, 6, 25). Together, these data revealed that matrix detachment leads to rapid and sustained activation of AMPK.

To test whether the rapid activation of AMPK upon matrix deprivation is cell line-specific, we took cancer cell lines from different tissues, such as breast (MCF7), cervix (HeLa S3), lung (A549), melanoma (G361) and human embryonic kidney (HEK 293T), and subjected them to detachment (suspension culture) for 10 min. All of the tested cell lysates showed an increase in the levels of pAMPKα under detached conditions (Fig. 1*B*). Total AMPK levels remained unchanged (Fig. 1*B*). To further confirm whether the increase in phosphorylation of AMPKα Thr-172 also corresponds with an increase in its activity, we measured the phosphorylation of its downstream target, ACC (26). We observed that the levels of pACC (Ser-79) were correspondingly increased under detached conditions in all these cell lines (Fig. 1*B*). This suggested that the rapid activation of AMPK upon detachment is not a cell line-specific phenomenon.

To further confirm the rapid activation of AMPK upon detachment, we undertook various approaches to gauge AMPK activity in cells cultured under attached and detached conditions. For most assays on rapid activation of AMPK, we chose to subject cells to 10 min of suspension culture following trypsinization prior to harvesting, unless mentioned otherwise. Immunocytochemical analysis revealed higher intensity of signal for both pAMPKα and pACC under detached conditions (Fig. 1*C*). In addition, we performed *in vitro* kinase assay with AMPK immunoprecipitated from cells grown under both attached and detached conditions using AMARA as the substrate peptide (27). We observed an almost 10-fold higher AMPK activity under detached conditions compared with attached culture (Fig. 1*D*). In yet another independent assay, cells stably expressing the AMPK activity reporter FRET construct AMPK-AR (17) were cultured under attached and detached conditions, and FRET ratio was measured. We detected a higher FRET ratio in detached conditions indicating higher AMPK activity (Fig. 1*E*). Thus, these data confirmed rapid AMPK activation within 10 min upon cell detachment.

To address whether AMPK activation was dependent on any specific mode of detachment, because trypsin treatment also leads to the cleavage of several surface receptors besides causing cell detachment, we used multiple approaches to detach cells, including cell dissociation using TrypLE (28) and mechanical detachment (19, 21, 22). pAMPKα levels were observed to be higher in detached cells compared with attached conditions in all the different modes tested (Fig. 1*F*). Thus, AMPK activation under detached conditions is likely to be independent of the mode of detachment.

To understand whether matrix detachment-triggered AMPK activation reverses when cells re-attach, we allowed detached cells to re-attach on tissue culture dishes. Both immunoblotting (Fig. 1*G*) and immunocytochemistry (Fig. 1*H*) confirmed that indeed AMPK activation is reversed on re-attachment (Fig. 1, *G* and *H*). Taken together, these data revealed that AMPK activation following cell detachment occurs rapidly and is independent of the mode of detachment or cell type, whereas matrix attachment leads to its inactivation.

##### AMPK Phosphorylation Is Independent of FAK and Src Signaling

**FIGURE 2.**
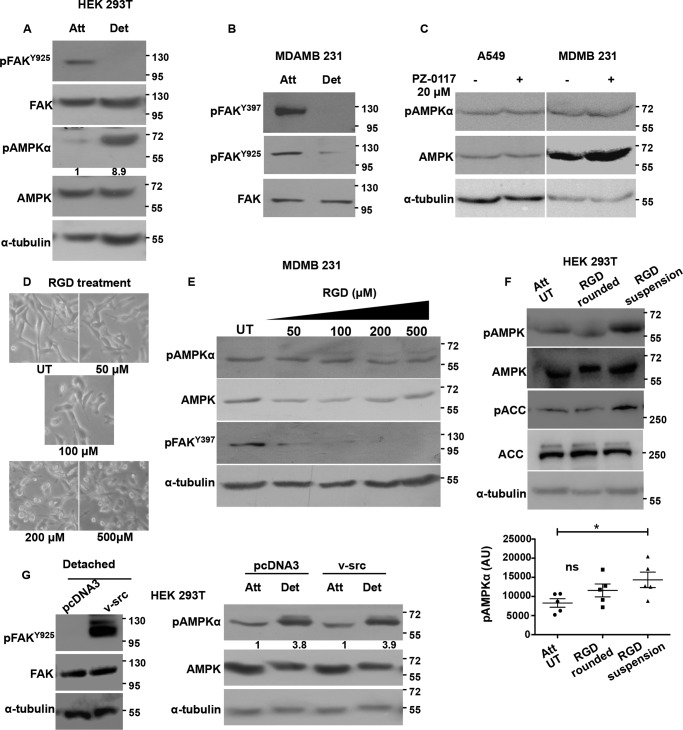
**AMPK phosphorylation is independent of FAK and Src signaling.** HEK 293T cells (*A*) and MDA-MB 231 cells (*B*) were cultured under attached (*Att*) and detached (*Det*) (10 min) conditions, and levels of indicated total proteins and phosphoproteins were determined by Western blotting (*n* = 3). *C,* A549 and MDA-MB 231 cells were cultured under attached conditions and treated for 1 h with FAK inhibitor PZ-0117 (20 μm). pAMPKα and AMPK levels were measured by Western blotting (*n* = 3). *D,* MDA-MB 231 cells cultured under attached conditions were treated with RGD peptide at the concentrations specified for 2 h. Phase contrast images for cell morphology changes were taken at 2 h post-treatment with RGD, and *E,* cells were lysed and pAMPKα, AMPK, and pFAK^Tyr-397^ levels were measured by Western blotting (*n* = 5). *F,* HEK 293T cells were treated with RGD (200 μm) for 10 min (leading to cell rounding) or 30 min (leading to cell detachment). Cells were lysed, and pAMPKα, pACC, total AMPK, and ACC were determined by Western blotting (*n* = 5). The scatterplot depicts raw pAMPK densitometric values normalized to the relative tubulin levels in each experiment .*, *p* < 0.05. *Error bars* represent ± S.E. *G,* HEK 293T cells were transfected with vector control pcDNA3 or constitutively active v-Src construct. After 24 h, the transfected cells were then cultured in attached or detached conditions. Phosphorylation of FAK by v-Src at Tyr-925 was determined by Western blotting under detached conditions. Levels of pAMPKα and AMPK were probed by Western blotting under attached and detached conditions (*n* = 3). *Numbers* depict relative pAMPKα/AMPK ratio. *ns*, non-significant; *AU*, arbitrary units; *UT*, were treated with vehicle.

Because detachment leads to a rapid loss of integrin signaling (29) and concomitant AMPK activation, we explored a possible negative cross-talk between integrin signaling and AMPK. We used integrin-induced autophosphorylation of FAK at tyrosine 397 (pFAK^Tyr-397^) or its phosphorylation by Src at tyrosine 925 (pFAK^Tyr-925^) as a measure of integrin signaling (30). We found that although AMPK was active (as indicated by pAMPKα and pACC levels) under detached conditions, pFAK^Tyr-397^ as well as pFAK^Tyr-925^ levels were significantly reduced under detached conditions in HEK 293T (Fig. 2*A*) and MDA-MB 231 (Fig. 2*B*) cells. Total FAK levels remained similar under the two conditions. These results confirmed that under attached conditions, when integrin signaling is high, AMPK activity is low, although the reverse holds true in suspension.

To understand whether integrin signaling has a causal role to play in the modulation of AMPK phosphorylation, we inhibited FAK with PZ-0117 (31). We found a corresponding reduction in autophosphorylation of FAK (pFAK^Tyr-397^) upon PZ-0117 treatment (data not shown). However, we did not find a corresponding increase in pAMPKα as would be expected if integrin signaling through FAK were to contribute to a reduction in AMPK phosphorylation (Fig. 2*C*). To further explore the role of integrin signaling in regulating AMPK activity, we partially inhibited the pathway using soluble arginine-glycine-aspartic acid (RGD) peptide (32). Addition of RGD to cells for 1 h caused a reduction in pFAK^Tyr-397^ as well as cell rounding in MDA-MB 231 cells at higher concentrations (Fig. 2, *D* and *E*). However, under these conditions, we could not detect any increase in pAMPKα levels (Fig. 2*E*). Thus, negative modulation of integrin functions under adherent conditions failed to bring about AMPK activation. We undertook the same experiment in another cell type HEK 293T, where RGD (200 μm) treatment caused cells to round up within 10 min, and by 30 min most of the cells had detached. Immunoblotting for pAMPKα and its downstream read-out pACC revealed that cell rounding did not activate AMPK; however, cell detachment induced by RGD could activate AMPK (Fig. 2*F*). These results suggested that AMPK activation under detached conditions could be independent of reduction in integrin signaling.

To further address the relationship between integrin signaling and AMPK, we induced integrin signaling in suspended cells using a genetic approach and measured the status of AMPK signaling in suspension. To do so, we transfected HEK 293T cells with either empty vector or constitutively active Src kinase (v-Src)-expressing vector. The v-Src-expressing cells when cultured in detached conditions showed high pFAK^Tyr-925^levels over vector control suggesting that these cells have elevated FAK-Src signaling in detached conditions (Fig. 2*G*). Interestingly, when we cultured HEK 293T cells expressing vector control or v-Src constructs in attached and detached conditions and probed for pAMPKα in these lysates, we did not observe any change in its levels between vector control and v-Src-transfected cells, both under attached (*1st* and *3rd lanes*) and detached conditions (*2nd* and *4th lanes*) (Fig. 2*G*). Thus, the detachment-induced increase in AMPK activity was unperturbed by constitutively active v-Src. Taken together, these results indicated that AMPK modulation between attached and detached conditions might be independent of integrin signaling through FAK and Src kinases.

##### Upstream Kinases LKB1 and CaMKKβ Play a Role in Detachment-induced AMPK Activation

Upstream kinases play an important role in activating AMPK (12). While LKB1 is implicated as the major kinase upstream of AMPK under several stress conditions, CaMKKβ is shown to play a major role in AMPK activation in response to elevated calcium levels. We next explored the roles of these two major upstream kinases in matrix deprivation-triggered AMPK activation.

**FIGURE 3.**
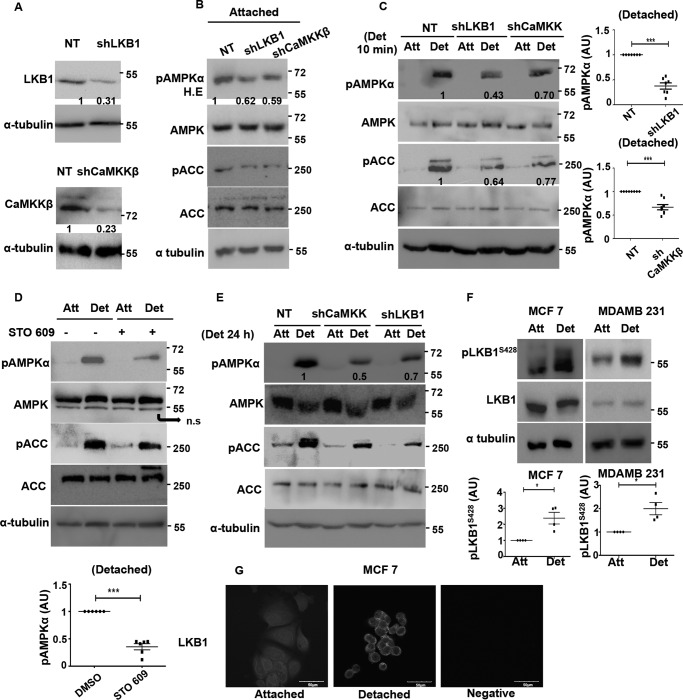
**Role of upstream kinases in detachment-induced AMPK activation in LKB1 containing MDA-MB 231 cells.** *A,* MDA-MB 231 cells stably expressing pGIPZ non-targeting shRNA (*NT*) or shRNA targeting LKB1 (*shLKB1*) or shRNA targeting CaMKKβ (*shCaMKK*β) were harvested for immunoblotting with LKB1 and CaMKKβ (*n* = 5). *Numbers* indicate relative LKB1/tubulin and CaMKKβ/tubulin ratio. *B,* MDA-MB 231 stably expressing NT, shLKB1, or shCaMKK were cultured under attached conditions. Levels of pAMPKα, pACC, AMPK, and ACC were measured by Western blotting (*n* = 3). The *numbers* depict relative pAMPKα/AMPK ratio. pAMPKα blot was obtained at high exposure (*H.E*). *C,* MDA-MB 231 cells expressing NT, shLKB1, or shCaMKKβ were cultured under attached (*Att*) and detached (*Det*) conditions. pAMPKα and pACC were measured by Western blotting (*n* = 7–8). *Numbers* indicate relative pAMPKα/tubulin ratio. The scatterplot depicts the fold change in pAMPKα/tubulin ratio of the knockdown cells relative to control NT cells; ***, *p* < 0.001. *Error bars* represent ± S.E. *D,* MDA-MB 231 cells were pretreated with STO-609, a specific inhibitor for CaMKKβ, for 2 h. Cells were then cultured under attached and detached (10 min) conditions, and pAMPKα, pACC, total AMPK, and ACC levels were measured by Western blotting (*n* = 6). The scatterplot depicts the fold change in pAMPKα/tubulin ratio; ***, *p* < 0.001. *Error bars* represent ± S.E. *ns,* non-significant. *E,* MDA-MB 231 cells stably expressing NT, shLKB1, or shCaMKKβ were cultured under attached and detached conditions for 24 h. pAMPKα and pACC were measured by Western blotting (*n* = 3). The *numbers* represent pAMPKα/tubulin ratio. *F,* MDA-MB 231 and MCF7 were cultured under attached and detached conditions. pLKB1 (Ser-428) and LKB1 levels were measured by Western blotting (*n* = 4). Scatterplot depict relative fold change in pLKB1/LKB1 ratio. *Error bars* represent ± S.E. *G,* MCF7 cells were cultured under attached and detached conditions, and immunocytochemistry was performed for LKB1. Representative confocal images of equatorial sections are shown. *AU*, arbitrary units.

The breast cancer cell line MDA-MB 231 expresses both LKB1 and CaMKKβ. To address the roles of LKB1 and CaMKKβ in AMPK activation upon detachment, we generated cell lines stably expressing knockdown constructs targeting LKB1 and CaMKKβ, respectively. We achieved around 75% knockdown of LKB1 and CaMKKβ in these cells (Fig. 3*A*). Knockdown of both the upstream kinases led to a reduction in the basal levels of pAMPK and pACC under adherent conditions (Fig. 3*B*) as well as under matrix deprivation (Fig. 3*C*), although the levels of total AMPK and ACC remained unchanged (Fig. 3, *B* and *C*). Additional independent shRNA sequences targeting LKB1 and CaMKKβ also showed a reduction in AMPK activity upon detachment (data not shown). We also obtained similar reduction in pAMPKα levels upon detachment with the CaMKKβ-specific inhibitor, STO-609 (Fig. 3*D*) (33). Together, these data suggested that in MDA-MB 231 cells, both LKB1 and CaMKKβ contribute to rapid AMPK activation following cell detachment. To investigate whether both of these kinases also contributed to sustained AMPK activation in suspension, we subjected pGIPZ NT (non-targeting shRNA)-, shLKB1-, and shCaMKKβ-expressing MDA-MB 231 cells to 24 h of suspension culture. Yet again, knockdown of either LKB1 or CaMKKβ led to a reduction in pAMPK levels (Fig. 3*E*). Thus, our data indicated that both upstream kinases LKB1 and CaMKKβ contribute to rapid and sustained activation of AMPK signaling under detached conditions in MDA-MB 231 cells.

LKB1 phosphorylation and its cytosolic localization is known to favor AMPK phosphorylation (34). Therefore, we next asked whether LKB1 phosphorylation and/or localization is modulated by detachment. We probed for Ser(P)-428 on LKB1 in both MDA-MB 231 and MCF7 cells. We observed a rapid increase in LKB1 phosphorylation in both cell types (Fig. 3*F*); total LKB1 remained unchanged. LKB1 phosphorylation at Ser-428 is known to cause its nuclear to cytoplasmic translocation (34, 35). We performed immunocytochemistry with LKB1 antibody on attached and detached cells. Interestingly, we observed that there is a nuclear to cytoplasmic translocation of LKB1 in detached conditions (Fig. 3*G*). These together suggest that matrix deprivation might trigger AMPK activation in part through the phosphorylation and translocation of LKB1.

**FIGURE 4.**
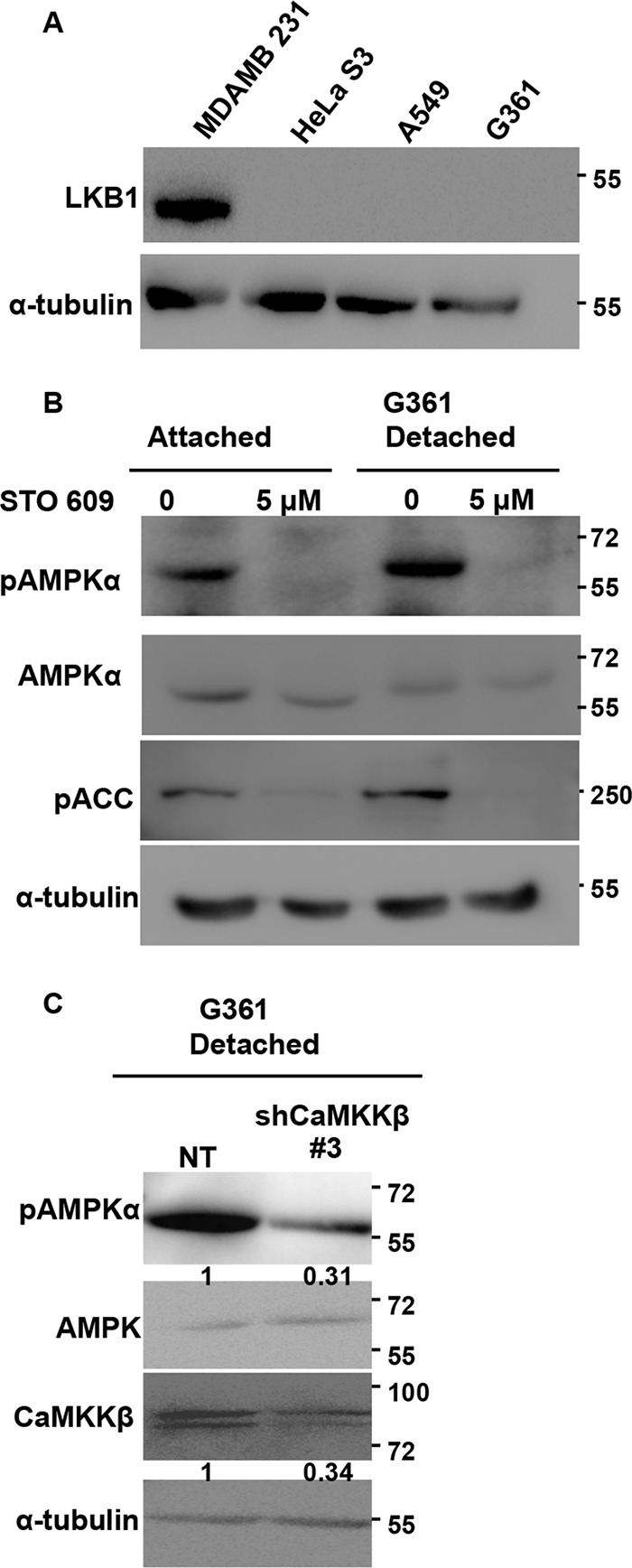
**Role of upstream kinases in detachment-induced AMPK activation in LKB1-deficient G361 cells.** *A,* multiple cell lines were cultured under attached conditions, and LKB1 levels were measured by Western blotting. *B,* G361 cells were pretreated with STO-609 and cultured under attached and detached conditions. pAMPKα and pACC levels were measured by Western blotting. The *numbers* represent relative pAMPKα/tubulin ratio. *C,* G361 cells were transfected with pGIPZ non-targeting shRNA (*NT*) or shRNA targeting CaMKKβ (*shCaMKK*β #*3*). Cells stably expressing these constructs were cultured under detached conditions for 10 min, and the levels of pAMPKα, CaMKKβ, and AMPK were measured by Western blotting (*n* = 4). The *numbers* depict relative pAMPKα/AMPK and CaMKKβ/tubulin ratios.

In the panel of cell lines used in this study, three cell lines, G361, A549, and HeLa S3, are known to be LKB1-deficient, and we confirmed the same by immunoblotting (Fig. 4*A*). As seen previously, these cell lines are also capable of rapidly activating AMPK upon detachment (Fig. 1*B*). Hence, we assessed the role of CaMKKβ in matrix deprivation-induced AMPK activation in the LKB1-deficient cells. Treatment of LKB1-deficient G361 cells with STO-609 led to almost complete loss of detectable pAMPK both under adherent and detached conditions (Fig. 4*B*). Similar results were obtained for A549, another LKB1-deficient cell line, in the presence of STO-609 (data not shown). Furthermore, G361 cells stably expressing shRNA against CaMKKβ (Fig. 4*C*) also showed a dramatic decrease in pAMPK levels. Additional independent shRNA sequences targeting CaMKKβ also showed a reduction in AMPK activity in detachment (data not shown), suggesting that in LKB1-deficient cells, under detached conditions, CaMKKβ is likely the major upstream kinase activating AMPK.

##### Calcium Signaling Contributes to AMPK Activation upon Detachment

**FIGURE 5.**
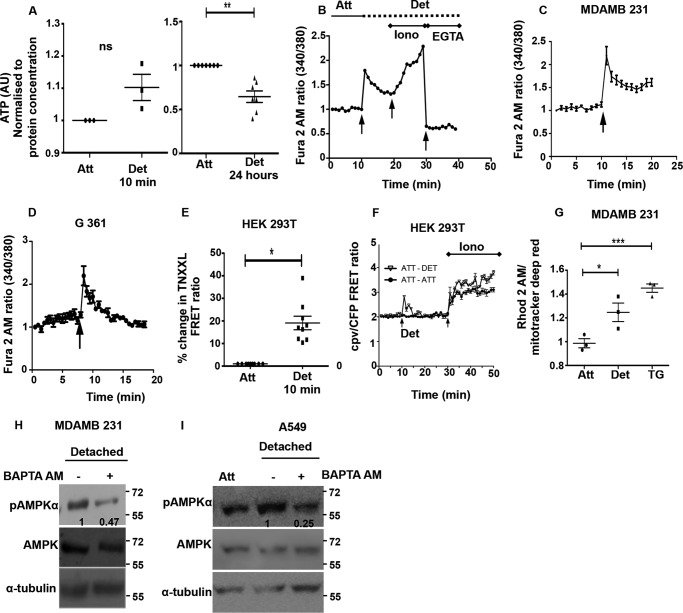
**Calcium signaling contributes to AMPK activation upon detachment.** *A,* HEK 293T cells were cultured under attached (*Att*) and detached (*Det*) conditions and lysed at 10 min or 24 h following detachment as indicated. ATP levels were measured using ATP Bioluminescence assay kit (*n* = 3–7). The values were normalized to protein concentration in the lysate. *Error bars* represent ± S.E.; *ns* = not significant; **, *p* < 0.01. *B,* cells were loaded with Fura 2 AM, a ratiometric dye for calcium measurements. *Graph* depicts representative time course measurements of emission obtained at 510 nm by excitation of the dye at 340 and 380 nm (340:380 ratio). The *1st black arrow* represents the point of mechanical detachment in the time course. Values have been normalized to the initial reading. The *2nd black arrow* represents the point of addition of positive control ionomycin (*Iono*). The *3rd black arrow* represents addition of EGTA along with 1% Triton X-100 as a negative control. MDA-MB 231 cells (*C*) and G361 cells (*D*) were loaded with Fura 2 AM, and the assay was performed as described in *B* (*n* = 4 independent experiments with three technical repeats in each experiment). Values depict mean ± S.E. *Arrow* represents the point of mechanical detachment. *E,* HEK 293T cells were transfected with calcium-sensitive FRET construct TN-XXL and sorted to obtain a population of cells with high expression of the FRET construct. These cells were cultured in attached conditions and detached mechanically. Intracellular calcium was measured in a plate reader format. Scatterplot depicts % change in the FRET ratio (CY/CC) normalized to attached condition (set to 1). *Error bars* represent ± S.E.; *, *p* < 0.05 (*n* = 9). *F,* HEK 293T cells were transfected with 4mtD3cpv, and FRET ratio of Venus emission (530 nm) to cyan emission (470 nm) was calculated on 430 nm excitation in attached (*Att*) and detached (*Det*) cells as a time course. The *1st arrow* represents the point of mechanical detachment, and the *2nd arrow* indicates the addition of ionomycin (*Iono*) (*n* = three independent experiments with three technical repeats). *Solid black circle* represents values from cells that remained attached throughout the time course, and *inverted triangle* represents values from cells detached at 10 min. *G,* MDA-MB 231 cells were loaded with Rhod 2-AM (10 μm for 30 min), a mitochondrial calcium-sensing dye. The scatterplot depicts peak calcium under detached conditions compared with baseline attached conditions (*n* = 3). Each point represents an average of three technical repeats *, *p* < 0.0; ***, *p* < 0.001. Thapsigargin (*TG*) is used as positive control. *H,* MDA-MB 231 cells were loaded with BAPTA-AM, an intracellular calcium chelator for 30 min, and then cultured under detached conditions (10 min). Cells were lysed, and pAMPK and AMPK levels were measured by Western blotting. *Numbers* depict relative pAMPKα/AMPK ratio (*n* = 7); *I,* A549 cells were loaded with BAPTA-AM or vehicle control DMSO and then detached mechanically. Lysates were probed by Western blotting with the antibodies indicated. *Numbers* depict relative pAMPK/AMPK ratio (*n* = 3). *AU*, arbitrary units.

To understand the signals that might impinge on upstream kinases LKB1 and CaMKKβ upon matrix deprivation, we investigated the role of known modulators of AMPK activity. Increase in AMP levels promotes Thr-172 phosphorylation of AMPK by upstream kinases (37). Thus, stresses that lead to a depletion in ATP, and consequent increase in the AMP/ATP ratio, activate AMPK (37). To address whether the rapid increase in pAMPK levels within 10 min of detachment is a consequence of reduced ATP levels, we measured ATP using a bioluminescence assay kit (Sigma). Our results indicated that ATP levels remained unchanged in cells 10 min post-detachment (Fig. 5*A*). However, consistent with a previous report (5), a reduction in ATP levels was observed at 24 h (Fig. 5*A*). Therefore, the rapid activation of AMPK observed within 10 min may be independent of changes in cellular ATP levels.

One of the major mechanisms implicated in the literature for AMP-independent activation of AMPK is the calcium-CaMKKβ pathway (38). To investigate the possible role of calcium in the activation of AMPK upon detachment, we measured intracellular calcium levels in detached cells using the ratiometric dye Fura 2 AM (23). Because serine proteases such as trypsin are known to increase calcium levels independent of detachment (39), we have used mechanical detachment by cell scraping (19, 21, 22) instead of routine trypsinization for understanding the effects of cell detachment on the kinetics of calcium and its role in rapid AMPK activation. We undertook calcium measurements in a time course format using ionomycin and EGTA + 1% Triton X-100 at the end of the experiment as controls (Fig. 5*B*).

Interestingly, in MDA-MB 231 cells loaded with Fura 2 AM, we found that the ratio of fluorescence intensities of Fura 2 AM at 340/380 nm showed a rapid spike upon detachment (Fig. 5*C*). We observed a similar calcium spike in LKB1-deficient G361 cells subjected to detachment (Fig. 5*D*), together revealing that detachment leads to an increase in intracellular calcium levels in these cells. We confirmed this result further by using an alternative approach involving a FRET-based calcium sensor, TN-XXL (17). The FRET ratio was higher in detached cells compared with attached cells (Fig. 5*E*), indicating that detached cells have higher intracellular calcium levels. We also observed an increase in the FRET ratio of 4mtD3cpv (enhanced cyan fluorescent protein/Venus mitochondrial-targeted calcium biosensor) (40) on detachment (Fig. 5*F*). The fluorescence intensities of Rhod 2-AM (a mitochondrial calcium sensing dye) also increased in detached cells; thapsigargin was used as positive control (Fig. 5*G*). These data suggested that the increase in cytosolic calcium also leads to an increase in mitochondrial calcium. Importantly, in the presence of BAPTA-AM, a calcium chelator, we observed a reduction in pAMPK levels in detached MDA-MB 231 (Fig. 5*H*) and LKB1-deficient A549 cells (Fig. 5*I*) compared with untreated cells. Taken together, our results revealed a calcium spike upon detachment and suggested a role for calcium signaling in AMPK activation upon detachment.

##### ER Release of Calcium Coupled to Store-operated Calcium Entry Contributes to AMPK Activation upon Matrix Deprivation

**FIGURE 6.**
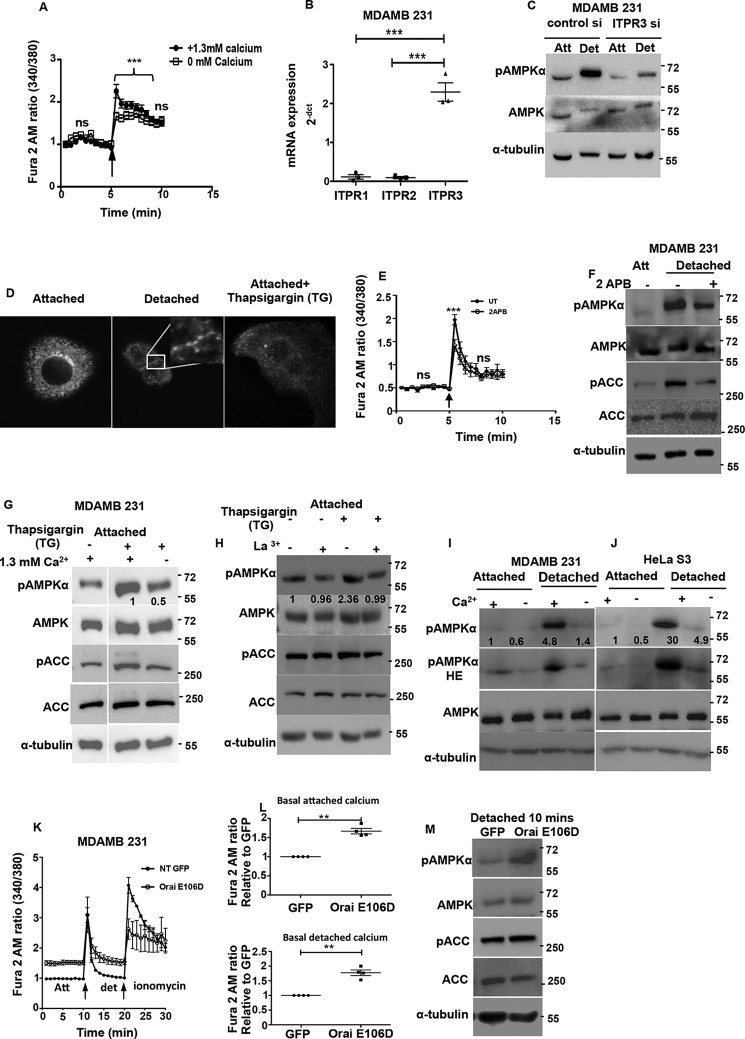
**ER calcium release and store-operated calcium entry contributes to AMPK activation upon matrix deprivation.** *A,* MDA-MB 231 cells were loaded with Fura 2 AM and cultured in calcium-containing or calcium-free buffer. *Graph* depicts time course measurements of emission obtained at 510 nm by excitation of the dye at 340 and 380 nm (340:380 ratio). The *black arrow* represents the point of mechanical detachment in the time course. Values have been normalized to the initial reading. Values are expressed as mean ± S.E.; ***, *p* < 0.001 (*n* = 4 with three technical repeats each). *ns,* non-significant. *B,* scatterplot depicts 2^−^*^dct^* values representing the mRNA expression of ITPR receptor subtypes determined by qRT-PCR (*n* = 3 biological samples with three technical repeats each). *C,* MDA-MB 231 cells were transfected with either control siRNA or ITPR3 siRNA and cultured under attached (*Att*) and detached (*Det*) conditions. Cell lysates were probed by Western blotting with the antibodies indicated. *D,* MCF7 cells were transfected with STIM1 YFP construct and cultured under attached and detached conditions. Representative confocal equatorial sections are shown. Thapsigargin (*TG*) was used as positive control. *E,* MDA-MB 231 cells were loaded with Fura 2 AM and pre-treated with either vehicle control (*UT*) or SOCE inhibitor 2-APB at 50 μm for 10 min. *Graph* depicts time course measurements of emission obtained at 510 nm by excitation of the dye at 340 and 380 nm (340:380 ratio). The *black arrow* represents the point of mechanical detachment in the time course. Values have been normalized to the initial reading. Values are expressed as mean ± S.E.; ***, *p* < 0.05 (*n* = 4 with three technical repeats each). *F,* MDA-MB 231 cells were cultured under attached conditions and pretreated with 100 μm 2-APB for 10 min prior to detachment. Cell lysates were subjected to Western blotting with the antibodies indicated (*n* = 3). *G,* attached MDA-MB 231 cells were treated with vehicle control or thapsigargin (*TG*) (200 nm) for 10 min in the presence or absence of extracellular calcium as indicated. Lysates were run together in the same gel and subjected to immunoblotting with the antibodies indicated (*n* = 3). *H,* attached MDA-MB 231 cells were treated with vehicle control or thapsigargin (200 nm) for 10 min in the presence or absence of La^3+^ (10 μm). Cell lysates were subjected to Western blotting for the antibodies indicated (*n* = 3). *I,* MDA-MB 231 cells were cultured in calcium-containing or calcium-free conditions for 30 min and detached as indicated for 10 min. Lysates were subjected to immunoblotting with the antibodies indicated (*n* = 3). *Numbers* depict relative pAMPKα/tubulin ratio. *J,* HeLa S3 cells were cultured in calcium-containing or calcium-free conditions for 30 min and detached as indicated for 10 min. Lysates were subjected to immunoblotting with the antibodies indicated (*n* = 3). *Numbers* depict relative pAMPKα/tubulin ratio. *K* and *L,* MDA-MB 231-GFP and MDA-MB 231 Orai E106D GFP cells were loaded with Fura 2 AM and cultured in calcium-containing KH buffer. *Graph* depicts time course measurements of emission obtained at 510 nm by excitation of the dye at 340 and 380 nm (340:380 ratio). The *black arrow* represents the point of mechanical detachment and ionomycin addition in the time course. Values are expressed as mean ± S.E.; **, *p* < 0.01 (*n* = 4 with 5 technical repeats each). *M,* MDA-MB 21 GFP and Orai E106D GFP cells were lysed under detached conditions, and lysates were subjected to Western blotting with the antibodies indicated.

We next investigated the mechanisms that might lead to the increase in intracellular calcium levels upon detachment. Within cells, ER is the major source of calcium. Calcium is released into the cytosol in response to various signaling events that cause opening of the inositol triphosphate receptors (ITPRs) on the ER membrane (store release) (41). To understand whether detachment leads to calcium release from the ER, we measured calcium changes using Fura 2 AM in detached cells. We observed an increase in intracellular calcium, both under calcium-containing and calcium-free (to prevent entry of extracellular calcium) buffer conditions (Fig. 6*A*) suggesting that ER release contributes to cytosolic calcium increase. To further gauge the involvement of ER calcium release in AMPK activation, we studied the major ER calcium release channels, the ITPRs. An RT-PCR analysis revealed that transcript levels of ITPR3 was severalfold higher than that of ITPR1 and -2 in MDA-MB 231 cells (Fig. 6*B*). Therefore, we undertook the siRNA approach to knock down ITPR3, and we found a significant reduction in pAMPK levels in matrix-deprived cells (Fig. 6*C*). This suggested that ER calcium release mediated by ITPR3 plays a role in AMPK activation upon matrix deprivation.

ER calcium sensors like STIM1 (42) sense loss of calcium from the ER lumen beyond a threshold, and this triggers extracellular calcium entry through the plasma membrane calcium channels into cells, a phenomena known as SOCE (43). As seen in Fig. 6*A*, the presence of extracellular calcium potentiated the increase in intracellular calcium levels upon detachment. When ER calcium levels are depleted, STIM1 is known to translocate close to the plasma membrane, oligomerize, and form puncta (43). To study the effect of matrix deprivation on STIM localization, we transfected MCF7 cells with the STIM-YFP construct (44). Treatment of adherent cells with thapsigargin (which causes ER calcium depletion through irreversible inhibition of the sarco/endoplasmic reticulum Ca^2+^-ATPase pump (45)) led to formation of STIM1 puncta (Fig. 6*D*). Interestingly, we observed similar punctate appearance of STIM1 under matrix deprivation (Fig. 6*D*), suggesting a possible role for SOCE in detachment-triggered intracellular calcium increase. To further confirm this, we additionally used 2-aminoethoxydiphenyl borate (2-APB), an inhibitor of SOCE (46), to prevent extracellular calcium entry upon cell detachment. Under these conditions, we observed a significant reduction in calcium peak upon detachment (Fig. 6*E*), as well as a reduction in pAMPK levels (Fig. 6*F*), together suggesting that the cytosolic calcium increase upon detachment might be due to intracellular store release coupled with extracellular calcium entry, which together lead to AMPK activation.

To further gauge whether SOCE is indeed important for AMPK activation, we used thapsigargin, which causes a depletion of ER calcium and subsequent induction of SOCE (47). As expected, thapsigargin treatment led to an increase in pAMPK levels under attached conditions in the presence of calcium-containing buffer. However, thapsigargin-induced AMPK activation was attenuated in calcium-free buffer (Fig. 6*G*). Similar attenuation of thapsigargin-induced AMPK activation was also observed in La^3+^ (SOCE inhibitor)-treated cells (Fig. 6*H*). This suggested that store-operated calcium entry likely plays a prominent role in AMPK activation. To further ascertain whether SOCE is indeed involved in detachment-induced AMPK activation, we compared pAMPK levels in cells detached in calcium-free and calcium-containing buffers. Interestingly, we observed that AMPK activation was impaired when MDA-MB 231 cells were detached in calcium-free buffer (Fig. 6*I*). We obtained similar results in LKB1-deficient HeLa S3 (Fig. 6*J*) and A549 cells (data not shown).

The store-operated calcium entry is facilitated by CRAC channels present on the plasma membrane (41). We investigated the effects of the E106D Orai1 calcium channel pore mutant, which leads to enhanced pore diameter (48). Interestingly, we observed that MDA-MB 231 cells stably expressing Orai E106D GFP had higher basal calcium levels compared with control GFP-expressing cells in both adherent and detached conditions (Fig. 6, *K* and *L*). We compared the levels of pAMPK in vector control and Orai E106D GFP stable cells under detached conditions. We found a significant increase in AMPK activation in cells expressing Orai E106D GFP (Fig. 6*M*). Taken together, our data indicate that detachment triggers calcium release from the ER and further leads to SOCE. Thus, calcium release from ER and extracellular calcium entry together contribute to rapid AMPK activation following detachment.

##### Oxidant Signaling Contributes to AMPK Activation

**FIGURE 7.**
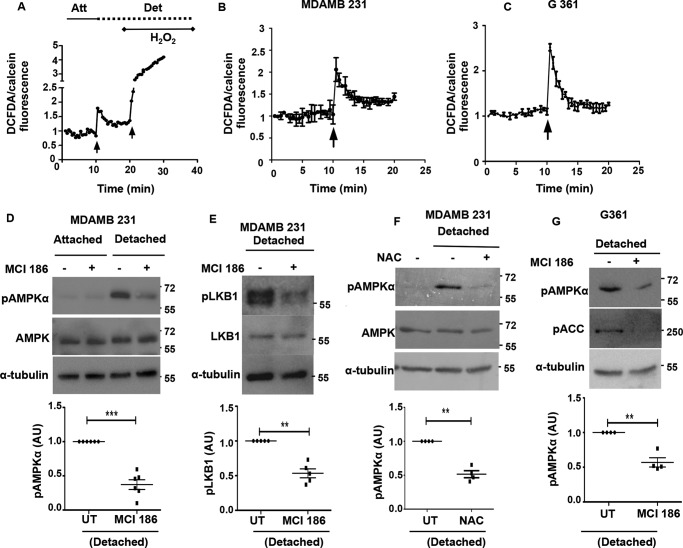
**Oxidant signaling contributes to AMPK activation upon matrix deprivation.** *A,* cells were loaded with DCFDA, a fluorescent dye for ROS measurements. Parallel wells were loaded with calcein AM as control. Fluorescence was measured at excitation 490/emission 520 nm in a spectrofluorometer in a plate reader format as a time course as represented in the *graph*. The *1st black arrow* represents the point of mechanical detachment in the time course. The *2nd black arrow* represents the addition of positive control H_2_O_2_. Fluorescence values were normalized to initial reading. DCFDA fluorescence/calcein fluorescence was calculated at each time point. *Att,* attached; *Det,* detached. MDA-MB 231 cells (*B*) G361 cells (*C*) were loaded with DCFDA, and ROS levels were measured as described above. The *black arrow* represents the point of mechanical detachment in the time course (*n* = 4 with three technical repeats each). Values are expressed as mean ± S.E. *D,* MDA-MB 231 cells pretreated with either vehicle control or MCI-186 (200 μm), for 2 h, were cultured under attached conditions or detached for 10 min, and Western blotting was performed to measure the levels of pAMPKα and AMPK (*n* = 6). The scatterplot depicts fold change in pAMPKα/tubulin ratio; ***, *p* < 0.001. *Error bars* represent ± S.E. *E,* MDA-MB 231 cells pretreated with either vehicle control or MCI-186 (200 μm), for 2 h, were detached for 10 min, and Western blotting was performed to measure the levels of pLKB1 Ser-428 and LKB1 (*n* = 5). The scatterplot depicts fold change in pLKB1/tubulin ratio; **, *p* < 0.01. *Error bars* represent ± S.E. *F,* MDA-MB 231 cells pretreated with either vehicle control or 1 mm NAC, for 2 h, were then either allowed to remain attached or detached for 10 min, and Western blotting was performed to measure the levels of pAMPKα and AMPK (*n* = 4). The scatterplot depicts fold change in pAMPKα/tubulin ratio; **, *p* < 0.01. *Error bars* represent ± S.E. *G,* LKB1-deficient G361 cells pretreated with either vehicle control or MCI-186 (200 μm), for 2 h, were then either allowed to remain attached or detached for 10 min, and Western blotting was performed to measure the levels of pAMPKα and AMPK (*n* = 4). The scatterplot depicts fold change in pAMPKα/tubulin ratio; **, *p* < 0.01. *Error bars* represent ± S.E.

We have previously observed a role for upstream kinase LKB1 in rapid AMPK activation upon detachment. Calcium signaling, however, is not known to impinge on LKB1. In contrast, in certain contexts, ROS levels are known to activate AMPK through LKB1 in the absence of changes in the AMP/ATP ratio (49). Hence, we investigated the role of endogenous oxidant signals as a possible regulator of AMPK activity upon matrix deprivation. To do so, we measured the levels of ROS using 2′,7′-dichlorofluorescein diacetate (DCFDA) (50) between attached and detached cells in a time course format using a fluorescence plate reader; treatment with H_2_O_2_ at the end of the experiment served as the positive control (Fig. 7*A*). To avoid the confounding effects of cell rounding and clumping on the fluorescence intensities, we loaded cells simultaneously with calcein-AM and used fluorescence measured from calcein-AM-loaded cells as a normalizing control. Yet again, because serine proteases such as trypsin are known to increase ROS levels independent of detachment (51), we have used mechanical detachment (19, 21, 22) instead of trypsin-EDTA for understanding the effects of cell detachment on the kinetics of oxidant signaling and its role in the observed rapid AMPK activation following detachment.

In MDA-MB 231 cells, measurement of ROS with DCFDA revealed an increase in oxidant signaling following detachment (Fig. 7*B*). A similar increase in ROS levels was also observed in LKB1-deficient cell lines like G361 following detachment (Fig. 7*C*). To understand whether the increase in ROS levels on detachment contributes to AMPK activation, we used the antioxidants 3-methyl-1-phenyl-2-pyrazolin-5-one (MCI-186) and *N*-acetyl cysteine (NAC) to quench cellular ROS levels by pretreatment of cells with these reagents before detachment. The ability of the antioxidants to quench ROS levels was confirmed by ROS measurements using DCFDA (data not shown). When MDA-MB 231 cells treated with MCI-186 were subjected to detachment, they showed reduced AMPK activation as gauged by reduced pAMPK levels (Fig. 7*D*), as well as a reduction in pLKB1 levels (Fig. 7*E*). Similarly, we obtained a reduction in pAMPK levels in the presence of yet another ROS quencher NAC (Fig. 7*F*). Interestingly, LKB1-deficient G361 cells, that are predominantly dependent on CaMKKβ for AMPK activation, also showed reduced AMPK activation upon treatment with antioxidants (Fig. 7*G*). This suggests that in LKB1-deficient cells ROS signaling might contribute to AMPK activation, perhaps working upstream of CaMKKβ. This is consistent with reports on the role of ROS in CaMKKβ-dependent AMPK activation in certain contexts (15, 52). Taken together, our results indicate that oxidant signaling triggered upon detachment also plays a role in AMPK activation.

##### Calcium-mediated Oxidant Signaling Contributes to AMPK Activation upon Detachment

**FIGURE 8.**
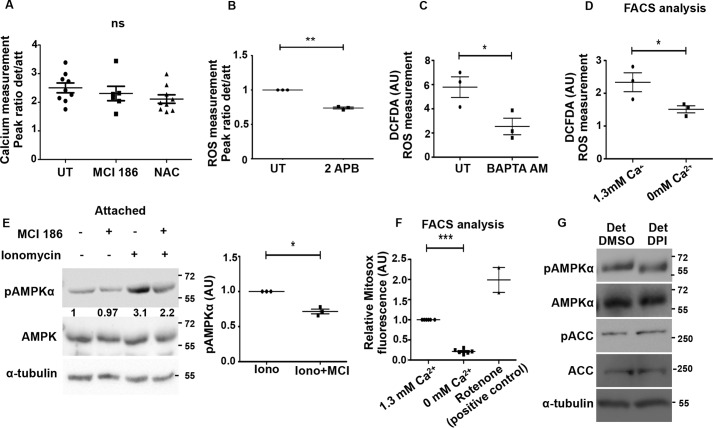
**Calcium mediates oxidant signaling upon detachment.** *A,* MDA-MB 231 cells were loaded with Fura 2 AM, a ratiometric dye for calcium measurements. Cells were pre-treated either with vehicle control or with MCI-186 (200 μm) or NAC (1 mm) as indicated. *Graph* depicts peak ratio (detached/attached) obtained from time course measurements carried out as described previously (*n* = 6–9). *Error bars* represent ± S.E.; *ns*, non-significant. *B–D,* MDA-MB 231 cells were pretreated with 2-APB (for 30 min) (*B*), BAPTA-AM (30 min) (*C*), or cultured in the presence or absence of calcium containing buffer for 30 min and subsequently loaded with DCFDA (for 10 min). Fluorescence intensities were measured under detached (*Det*) and attached (*At*t) conditions using spectrofluorometer as described previously (*B* and *C*). Detached cells were analyzed by FACS (*D*); **, *p* < 0.01; *, *p* < 0.05. *E,* adherent MDA-MB 231 cells were pretreated for 1 h with MCI-186 (200 μm) in the indicated lanes and subsequently treated with either vehicle control (DMSO) or ionomycin (1 μm) for 5 min. pAMPKα and AMPK levels were measured by immunoblotting (*n* = 3). *Numbers* represent relative pAMPKα/AMPK ratio. The scatterplot depicts relative fold change in pAMPK/tubulin ratio; *, *p* < 0.05. *Error bars* represent ± S.E. *F,* MDA-MB 231 cells were loaded with MitoSOX (2.5 μm) for 30 min and cultured in calcium-containing and calcium-free buffers. Cells were then detached mechanically and subjected to FACS analysis (*n* = 6). Rotenone, a mitochondrial electron transport inhibitor was used as positive control; ***, *p* < 0.001. *Error bars* represent ± S.E. *G,* MDA-MB 231 cells were treated with either vehicle control (*UT*) or diphenyleneiodonium chloride (*DPI*) and cultured under detached conditions. Cell lysates were probed by Western blotting with the antibodies indicated (*n* = 3).

As shown above, calcium and oxidant signaling are triggered immediately following detachment, and both contribute to AMPK activation upon detachment. These molecules may function in distinct or overlapping pathways upstream of the kinases LKB1 and CaMKKβ. To investigate whether there is a cross-talk between oxidant and calcium signaling under detached conditions, we first asked if ROS functions through calcium. ROS is known to cause calcium increase under hypoxia (15). To address this, we pre-treated MDA-MB 231 cells with the ROS quencher NAC or MCI-186 and compared the calcium surge on detachment with control untreated cells. We observed no significant change in the calcium peak on ROS inhibition (Fig. 8*A*), suggesting that calcium increase upon detachment is most likely independent of ROS signaling. Conversely, when we inhibited calcium surge using 2-APB, an inhibitor of extracellular calcium entry (Fig. 8*B*), BAPTA-AM, an intracellular calcium chelator (Fig. 8*C*), or by using calcium-free buffer to prevent SOCE (Fig. 8*D*), we observed a significant reduction in ROS levels. These results indicated that intracellular calcium increase contributes to ROS signaling upon detachment.

Our data for the first time revealed a calcium surge-dependent increase in ROS levels in detachment. To further confirm this signaling axis under attached conditions in MDA-MB 231 cells, we increased intracellular calcium levels using ionomycin, a known calcium ionophore (53). Increased cytosolic calcium levels triggered by ionomycin can activate AMPK (54). Consistent with this, we observed an increase in pAMPK levels in the presence of ionomycin (Fig. 8*E*). However, when we quenched ROS levels by pretreating the cells with MCI-186, ionomycin-mediated AMPK activation was partly abrogated (Fig. 8*E*) suggesting that calcium can impinge on AMPK activation, in part, through oxidant signaling.

Thus, in MDA-MB 231 cells, detachment leads to calcium increase, which in turn increases ROS, and together these contribute to AMPK activation through CaMKKβ and LKB1. These results suggest a novel mechanism involving calcium-dependent ROS signaling for the rapid AMPK activation under detached conditions.

##### Detachment-induced Calcium Surge Triggers Mitochondrial ROS

To address the source of ROS that could be sensitive to calcium changes, we measured mitochondrial ROS levels, which is known to contribute to AMPK activity in breast cancer cells (55). We measured mitochondrial superoxide levels using MitoSOX Red (56). Cells detached in calcium-containing buffer had higher levels of mitochondrial superoxide compared with cells detached in calcium-free buffer (Fig. 8*F*). Rotenone, an electron transport chain uncoupler, served as a positive control (Fig. 8*F*). Thus, our results indicate that detachment-induced increase in calcium could activate mitochondrial superoxide production, which might further cause AMPK activation. To further understand whether yet another source of ROS, the membrane NADPH oxidases, also contributes to AMPK activation, we inhibited the NADPH oxidases with diphenyleneiodonium. We observed no change in pAMPK levels (Fig. 8*G*) suggesting that the source of detachment-induced ROS is mitochondrial and not membrane-bound NOXs. Taken together, our results reveal a novel calcium-mediated AMPK activation in detachment driven in part by mitochondrial ROS.

##### LKB1/CaMKK-AMPK Axis Promotes Anchorage-independent Colony Formation in Cancer Cells

**FIGURE 9.**
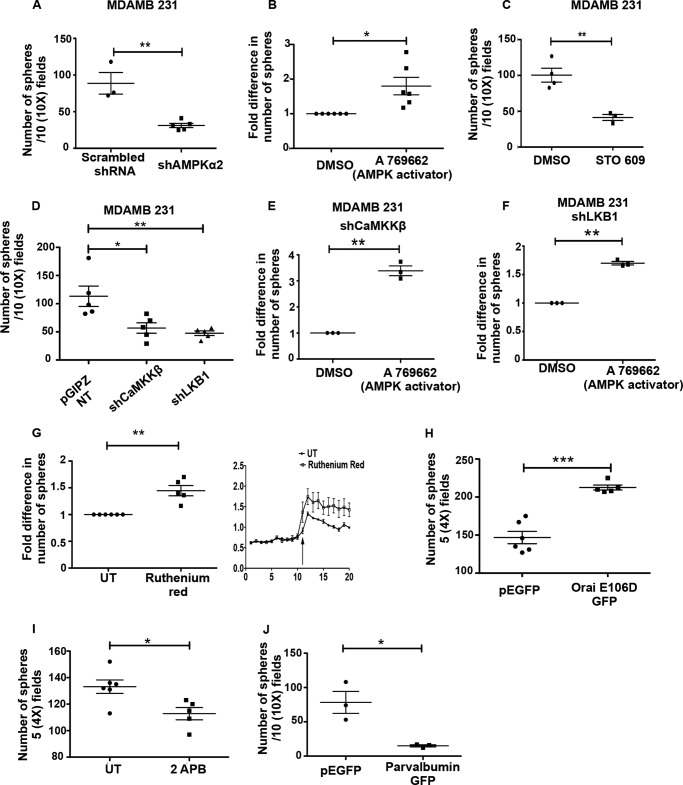
**AMPK signaling and intracellular calcium promotes sphere formation.** *A*, MDA-MB 231 cells expressing either scrambled shRNA or shRNA against AMPKα2 were subjected to anchorage-independent sphere formation assay. Numbers of spheres (having more than 10 cells) were counted from 10 independent random fields under ×10 magnification. The scatterplot represents results from at least three independent experiments with two technical replicates each. *Error bars* represent ± S.E.; **, *p* < 0.01. *B,* MDA-MB 231 cells treated with DMSO (vehicle control) or A769662 were subjected to anchorage-independent colony formation. The scatterplot represents relative fold change from three independent experiments with two technical replicates each. *Error bars* represent ± S.E.; *, *p* < 0.05. *C,* MDA-MB 231 cells were treated with STO-609 and subjected to anchorage-independent sphere formation assay. *Graph* represents results from three independent experiments. *Error bars* represent ± S.E.; **, *p* < 0.01. *D,* MDA-MB 231 NT, shLKB1, and shCaMKKβ cells were subjected to anchorage-independent sphere formation assay and quantified as described above. The scatterplot represents the number of spheres from five independent biological sets; **, *p* < 0.01; *, *p* < 0.05. *Error bars* represent ± S.E. *E* and *F,* MDA-MB 231 shCaMKKβ cells (*E*) and shLKB1 cells (*F*) were treated with DMSO or A769662 and subjected to anchorage-independent sphere formation assay and quantified as described above. *Error bars* represent ± S.E.; **, *p* < 0.01. *G,* MDA-MB 231 cells treated with either vehicle control (*UT*) or ruthenium red (25 μm) were subjected to anchorage-independent sphere formation assay and quantified as described above. *Error bars* represent ± S.E.; **, *p* < 0.01. MDA-MB 231 cells were loaded with Fura 2 AM, and calcium measurements were carried out as described under “Experimental Procedures” in UT and ruthenium red-treated cells. *H,* MDA-MB 231 cells expressing either GFP or Orai E106D GFP were subjected to anchorage-independent sphere formation assay and quantified as described above. Number of spheres were counted in five random ×4 fields per dish. *Graph* represents results from three independent experiments with three technical replicates each. *Error bars* represent ± S.E.; ***, *p* < 0.001. *I,* MDA-MB 231 cells were treated with either vehicle control (*UT*) or 2-APB (100 μm) and were subjected to anchorage-independent sphere formation assay and quantified as described above. *Error bars* represent ± S.E.;*, *p* < 0.05. *J*, MDA-MB 231 cells expressing either GFP or parvalbumin GFP were subjected to anchorage-independent sphere formation assay and quantified as described above. Number of spheres were counted in 10 random ×10 fields per dish. Graph represents results from three independent experiments. *Error bars* represent ± S.E.; *, *p* < 0.05.

Recent work from our laboratory (6) and that of others (7, 8) showed the importance of AMPK in anchorage-independent colony formation in breast cancer cells. To understand the role of upstream kinases in this process, we undertook sphere formation assays in soft agar under conditions of inhibition or knockdown of CaMKK and LKB1. Consistent with our previous study (6), we found a reduction in sphere formation in MDA-MB 231 cells with AMPKα2 knockdown compared with cells expressing scrambled shRNA (Fig. 9*A*) and an increase in sphere formation in cells treated with the AMPK activator A769662 (Fig. 9*B*). Similarly, we observed that MDA-MB 231 cells formed fewer numbers of spheres on CaMKKβ inhibition with STO-609 (Fig. 9*C*). We obtained similar results in LKB1-deficient A549 cells treated with STO-609 (data not shown). Additionally, MDA-MB 231 cells expressing shCaMKKβ or shLKB1 also formed fewer numbers of spheres as compared with MDA-MB 231 NT cells (Fig. 9*D*). Furthermore, the reduction in colony formation of shCaMKKβ as well as shLKB1 cells was rescued by pharmacological activation of AMPK using A769662 (Fig. 9, *E* and *F*). Yet another AMPK activator, AICAR, also rescued the reduction in colony formation observed in shCaMKKβ cells (data not shown) suggesting that these upstream kinases might function through AMPK to promote anchorage-independent colony formation. These results together indicate that LKB1 and CaMKKβ might play a critical role in anchorage-independent colony formation through activation of AMPK.

Because our data identified the rise in intracellular calcium as a major mechanism of AMPK activation, we next sought to understand the role of calcium in sphere formation. To do so, we used multiple methods to modulate intracellular calcium levels. Using ruthenium red (25 μm), we could increase the detachment-induced calcium levels (Fig. 9*G*). This led to an increase in sphere formation potential in ruthenium-treated cells compared with untreated cells (Fig. 9*G*). We also used Orai E106D GFP stable cells, which showed increased basal calcium (Fig. 6*K*) under attached and sustained detachment. These cells also showed an increase in sphere formation (Fig. 9*H*). Treatment with 2-APB, which leads to a decrease in intracellular calcium (Fig. 6*E*), led to a decrease in sphere formation (Fig. 9*I*). Alternatively, we used MDA-MB 231 cells expressing parvalbumin nuclear exclusion signal GFP (Fig. 9*J*), which impairs cytosolic calcium signals (57). In this case, we found a dramatic reduction in the sphere formation ability of MDA-MB 231 cells. Taken together, these data identify a role for calcium and upstream kinases of AMPK in promoting anchorage-independent colony formation.

##### Increased LKB1 and pACC Levels in Breast Cancer Patient Specimens

**FIGURE 10.**
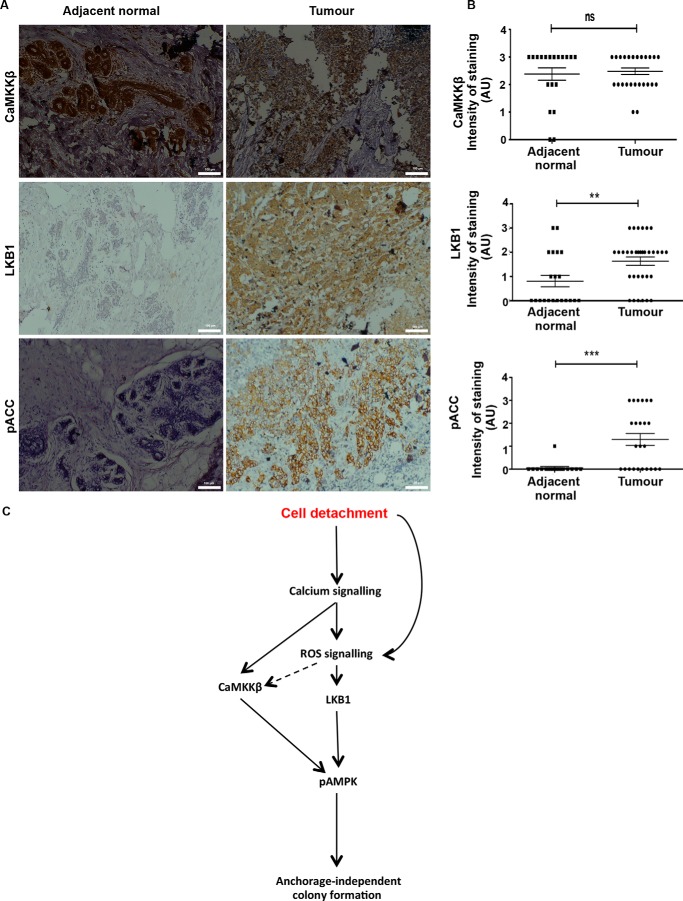
**AMPK signaling is active *in vivo* in breast tumors.** *A* and *B,* cancer breast tissue samples and adjacent normal tissue sections were subjected to immunohistochemical staining as described under “Experimental Procedures” for CaMKKβ, LKB1, and pACC. *A,* representative bright field images (×10 magnification) of IHC on both tumor and normal samples. *B, graph* depicts semiquantitative intensity values of staining for the mentioned antibody graded from 0 to 3 for both normal and tumor samples. *ns,* non-significant; **, *p* < 0.01; ***, *p* < 0.001. *C,* cell detachment from the matrix rapidly triggers calcium and oxidant signaling. Calcium signals potentiate the levels of ROS. Together, these signaling molecules impinge on AMPK signaling pathway through the upstream kinases LKB1 and CaMKKβ. Activation of AMPK under matrix deprivation promotes anchorage-independent colony formation.

To address the relevance of the LKB1/CaMKK-AMPK axis *in vivo*, we undertook immunohistochemistry on tissue sections from grade III invasive carcinoma of breast compared with adjacent normal tissue. As shown in Fig. 10*A,* we detected high levels of CaMKKβ expression, which was not significantly altered between normal and tumor samples. Interestingly, and in contrast to previous reports on breast cancer (58, 59), we found an increase in LKB1 levels in breast cancer samples compared with adjacent normal tissue (Fig. 10, *A* and *B*). We used pACC as a read-out for AMPK activity, and consistent with a recent study (55), we found it to increase significantly in invasive breast cancers compared with adjacent normal tissue (Fig. 10, *A* and *B*). We also observed a significant correlation between LKB1 and pACC in the cancer samples (*p* = 0.0104; Fisher's exact test). Together, these results suggest that LKB1/CaMKKβ-AMPK pathway activation could potentially contribute to tumor progression.

## Discussion

Recent work from our laboratory (6) and that of others (7, 8, 25) has demonstrated AMPK activation upon matrix deprivation and its role in anoikis resistance. However, the molecular mechanisms that lead to AMPK activation upon matrix deprivation are not well delineated. One of the major mechanisms of AMPK activation is energy stress caused by reduced ATP levels (12). Although matrix deprivation causes ATP depletion by 24 h (5), our data revealed that AMPK is activated very early, within minutes, following detachment. In this study, we show a rapid increase in calcium and ROS levels following cell detachment, and we identify a novel calcium-redox signaling network that leads to rapid AMPK activation upon matrix detachment through the upstream kinases LKB1 and CaMKKβ (Fig. 10*C*). We speculate that this early activation of AMPK could help prepare the cells to face the imminent energy crisis that follows sustained ECM detachment.

LKB1 and CaMKKβ are two well studied upstream kinases that are known to activate AMPK under different stress conditions. Although LKB1 is majorly known to activate AMPK under energetic stress, CaMKKβ is implicated under conditions of increased cytosolic calcium independent of the AMP/ATP ratio (60). In this study we find that upstream kinases LKB1 as well as CaMKKβ play important roles in AMPK activation upon matrix detachment in MDA-MB 231 cells, although CaMKKβ is the major player in this context in LKB1-deficient cell lines like G361 and A549. However, one cannot rule out the contributions of other recently identified AMPK kinases such as TAK1, ATM, and MLK3 (61, 62) as well as phosphatases, that may help to fine-tune AMPK activity in suspension; their roles in AMPK activation in matrix-deprived cells need to be explored in the future.

Calcium and ROS are ubiquitous second messengers that mediate various physiological functions. Although calcium is known to activate AMPK through CaMKKβ (54), oxidant signaling is reported to activate AMPK through LKB1 (49). Our study demonstrates for the first time a rapid increase in calcium and oxidant signaling upon detachment. This increase in calcium and ROS contributes to the rapid activation of AMPK upon detachment. Indeed, oxidant signaling is shown to rapidly induce calcium signaling, which further activates CaMKKβ-AMPK axis under hypoxia (15, 52). In our study, however, we find that calcium signaling is upstream of oxidant signals in matrix-deprived MDA-MB 231 cells. Strikingly similar observations have been made in epidermal wounding response where calcium functions upstream of ROS signals (63). Calcium-ROS signaling network, each potentiating the other, have also been reported downstream of B cell receptor signaling (64). Thus, these second messengers seem to regulate each other in a context-dependent manner. We further demonstrate that under detached conditions calcium influx triggers mitochondrial ROS, which is known to activate AMPK in breast cancers cells (55) and also in response to hypoxia (52). These results together suggest that mitochondrial ROS production in response to a rise in intracellular calcium could trigger AMPK activation upon detachment. Thus, our study provides novel insights into detachment-induced calcium and oxidant signals and their role in regulating AMPK in the context of matrix deprivation. Calcium and ROS signals may additionally regulate a plethora of proteins enabling cells to mount an effective response to the stress of ECM deprivation.

Our data showed that ER calcium release and the associated SOCE upon matrix deprivation plays a critical role in the process of AMPK activation. We further showed that the increase in intracellular calcium promotes anchorage-independent sphere formation. However, how matrix deprivation triggers ER-calcium release remains to be addressed. One possibility is that matrix deprivation, which is known to activate PERK (65), an ER resident channel, might cause ER calcium release. Yet another possibility is that cytoskeletal changes caused by detachment (18) may trigger ER calcium release as well as SOCE. Indeed, inhibition of microtubule polymerization by nocodazole causes STIM1 translocation and SOCE (66). Currently, however, a clear understanding of how matrix deprivation leads to ER calcium release is lacking.

ROS can function as a signaling molecule (oxidant signaling) in the maintenance of physiological functions or in elevated levels (oxidative stress) can cause damage to cellular proteins, lipids, and DNA (67). Although our data revealed a role for ROS in the rapid activation of AMPK following detachment, sustained ECM deprivation is known to lead to oxidative stress (65), and AMPK is known to mitigate the same (8). Together, these observations suggest that the initial increase in ROS signaling in response to detachment could initiate AMPK activation, which could in turn bring about redox homeostasis during prolonged ECM deprivation; this hypothesis needs to be further explored.

In our studies, we report that AMPK is rapidly dephosphorylated when cells reattach to the culture dish. Multiple studies have shown that integrin engagement to ligand, which takes place upon attachment, can increase intracellular calcium levels (68, 69). We therefore speculate that AMPK inhibition upon re-attachment could be brought about by a calcium-independent regulatory system. We observed that the reattachment to collagen and fibronectin also reduced pAMPK levels rapidly (data not shown). Because AMPK dephosphorylation occurs rapidly following attachment, one possibility is rapid dephosphorylation of AMPK by phosphatases like PP2A, PP2C, or PpmIE (70). Yet another possibility is the dephosphorylation of upstream kinase LKB1 and possible nuclear shuttling of the kinase upon cell reattachment. A clear cause of how matrix attachment leads to AMPK inactivation needs to be worked out.

The LKB1-AMPK axis is regarded typically as a tumor suppressor pathway (71). However, the CaMKKβ-AMPK axis is ascribed many tumor-promoting roles. For example, this pathway has recently been implicated in the migration of prostate cancer cells (72). Furthermore, CaMKKβ has been shown to have a pro-tumorigenic role in androgen-dependent prostate cancers with androgens directly inducing CaMKKβ expression at mRNA and protein levels (73). Interestingly, our data revealed that both LKB1 and CaMKKβ played a positive role in the anchorage-independent colony formation assay in MDA-MB 231 cells. However, contrary to reported literature (58, 59, 74), our study shows LKB1 overexpression in several invasive ductal carcinoma patient samples compared with adjacent normal tissue. LKB1 expression correlated with pACC in these samples suggesting that the LKB1-AMPK axis could be active in some invasive grade III tumors where it could potentially play a role in tumor promotion. LKB1 mutations are reported to be rare in breast cancers unlike non-small cell lung carcinoma (NSCLC) and colon cancers (75). In late stage hepatocellular carcinoma, LKB1 overexpression has been reported (76). LKB1 has been reported to promote survival of ovarian cancer spheroids (36). Our study corroborates these observations in other cancer types and points to a pro-tumorigenic role for the LKB1/CaMKKβ-AMPK pathway in breast cancer.

Thus, our study identifies a novel calcium-redox signaling network rapidly triggered by detachment that leads to AMPK activation through the upstream kinases LKB1 and CaMKKβ, and suggests that the Ca^2+^-ROS-CaMKK/LKB1-AMPK signaling axis might contribute to anoikis resistance and tumor progression.

## Experimental Procedures

### 

#### 

##### Cell Culture, Plasmids, Transfections, and Stable Cell Lines

Cell lines MDA-MB 231, HEK 293T, HeLa S3, MCF7, G361, and A549 (procured from ATCC) were cultured in DMEM (Sigma) with 10% fetal bovine serum (Invitrogen). All cell lines were maintained in standard 5% CO_2_ incubator at 37 °C. Plasmids encoding shRNA targeting LKB1 and CaMKKβ (pGIPZ backbone) as well as the non-targeting control shRNA, also in pGIPZ vector, were purchased from Dharmacon. Cells were transfected with the constructs pGIPZ NT, shLKB1, and shCaMKKβ using Lipofectamine 2000 (Invitrogen) and subsequently selected with puromycin (Sigma) to generate stable cells. AMPK-responsive FRET construct, AMPK AR (16), was a kind gift from Dr. Lewis Cantley. TN-XXL, a FRET construct for intracellular calcium, was a kind gift from Dr. Oliver Griesbeck (17). The construct pcDNA3-v-Src was a kind gift from Dr. Jungho Kim. 4mtD3cpv was a gift from Dr. Michael Davidson (Addgene plasmid 58184). Human STIM1-YFP and Orai 1 E106D Myc-GFP was a gift from Dr. Anjana Rao (Addgene plasmids 19754 and 22755).

##### Cell Detachment by Trypsinization

For experiments involving the study of the effect of various inhibitors or knockdown of upstream kinases on AMPK activation status, we have employed trypsinization as the mode of detachment (18–20). In brief, cells were trypsinized with 0.25% trypsin containing 0.9 mm EDTA (TE) from Thermo Scientific (catalog no. 25200-072), quenched in serum-containing media, and seeded on poly-HEMA (Sigma)-coated dishes for 10 min to 24 h. Cells were subsequently centrifuged and lysed for Western blotting.

##### Cell Detachment by Mechanical Scraping

For experiments involving modulation of ROS and calcium, we have used mechanical cell detachment (19, 21, 22). In brief, cells grown in 35-mm tissue culture dishes were mechanically scraped for 10 s with a rubber policeman (cell scraper) to bring cells to suspension in the same media (6 × 10^5^ cells/ml). Cells were incubated in suspension for 1–10 min as specified in each experiment and then collected into pre-chilled microcentrifuge tubes, centrifuged, and lysed for Western blotting.

For experiments involving the measurement of calcium and ROS in time course format using a spectrofluorometer, cells were grown in 96-well plates (2 × 10^4^ cells/well/100 μl). After taking basal fluorescence readings in adherent conditions (every 30 s for a period of 10 min), cells were mechanically detached by scraping using a pipette tip. In detached conditions, the time course format of taking readings every 30 s for 10 min was continued.

##### Western Blotting

Whole cell lysates for Western blotting were prepared with lysis buffer containing 1% Nonidet P-40 detergent, 0.5% sodium deoxycholate, 0.1% SDS, 50 mm sodium fluoride, 1 mm sodium orthovanadate, 10 mm sodium pyrophosphate (Sigma), and protease inhibitors (Roche Applied Science). Suspended cells were gently spun at 3000 rpm at 4 °C and lysed for Western blotting. Protein concentration was estimated with the Bradford reagent, and equal amounts of protein were resolved by SDS-PAGE and transferred to a PVDF membrane (EMD Millipore) and probed with appropriate antibodies. The membrane was incubated with horseradish peroxide (HRP)-coupled secondary antibodies (The Jackson Laboratory) and were developed using a PICO reagent (Thermo Fisher Scientific) in Syngene G box gel documentation system. Primary antibodies against pAMPK (catalog no. 2535), total AMPK (catalog no. 2532), phosphorylated acetyl-CoA carboxylase (pACC) (catalog no. 3661), total ACC (catalog no. 4190), and LKB1 (catalog no. 3050) are all of rabbit origin and were purchased from Cell Signaling Technology (Danvers, MA). The antibody against CaMKKβ (PA5-30558) was from Thermo Fisher Scientific. Anti-α-tubulin of mouse origin (catalog no. CP 06) (Calbiochem) served as the loading control in all Western blots.

##### Quantification of Western Blot

The intensity (area × optical density) of the individual bands on Western blots was measured by using ImageJ and normalized either to α-tubulin or, in the case of a phosphoprotein, to its total protein as mentioned. The graphs represent normalized values as fold change over control condition. One-sample Student's *t* test was performed to test for statistical significance. In Fig. 2*F*, a repeated measure one-way ANOVA is used.

##### Pharmacological Chemicals, Inhibitors/Activators

RGD, thapsigargin, and STO-609 were purchased from Calbiochem (Merck). DCFDA and PZ-0117 (FAK inhibitor) were from Sigma. Stock solutions were prepared in absolute dimethyl sulfoxide (DMSO, Calbiochem). BAPTA-AM, MitoSOX Red, and Fura 2 AM were purchased from Invitrogen. MCI-186 (sc-200806) was purchased from Santa Cruz Biotechnology.

##### Spectrofluorimetry for Calcium Measurements

Following trypsinization, 2 × 10^4^ cells were seeded into each well in a 96-well plate. After 24 h, 5 μm Fura 2 AM (a ratiometric dye) was loaded in Krebs-Henseleit (KH) buffer with 25 mm glucose, 1.3 mm calcium chloride, and 1 mm magnesium sulfate with 0.02% pluronic acid F-127 (Sigma) for 30 min at 37 °C (23). Cells were then given a wash with fresh buffer and incubated in it for 20 min for completion of esterase activity. The wells containing attached cells were measured at 340 and 380 nm fluorescence excitation with 510 nm emission for 5–10 min with readings taken every 30 s from at least five different regions in the well in a spectrofluorometer plate reader (Tecan Infinite M200 PRO). This served as the baseline calcium level for each well. In time course experiments, cells were detached gently by cell scraping with a pipette tip at the specified time. Following detachment, the readings were taken every 30 s for 10 min. The ratio of emissions at 340/380 nm indicates intracellular calcium levels. At the end of each experiment, positive and negative controls with ionomycin (1 μm) and EGTA (with 1% Triton X-100) were performed to determine the highest and lowest values obtainable with Fura 2 AM for each experiment. We have used two-way repeat measures ANOVA with Bonferroni's multiple comparison test for statistical analysis of differences in time course assays under different conditions.

##### Spectrofluorimetry and Fluorescence-activated Cell Sorting (FACS) for ROS Measurements

Following trypsinization, 2 × 10^4^ cells were seeded into each well in a 96-well plate. After 24 h, DCFDA was loaded in Krebs-Henseleit (KH) buffer with 25 mm glucose, 1.3 mm calcium chloride, and 1 mm magnesium sulfate for 15 min at 37 °C. Cells were then given a wash with fresh buffer, and the plates were measured at excitation 490/emission 520 nm for DCFDA. To normalize for the fluorescence intensity changes caused by cell rounding and clumping, we have used parallel wells loaded with calcein-AM and measured the same at excitation 490/emission 520 nm. In time course experiments, cells were detached gently by mechanical scraping. The ratio of fluorescence from DCFDA/calcein AM was plotted at each time point. For measuring ROS levels by FACS, 6 × 10^5^ cells in each 35-mm tissue culture dish were pretreated with requisite drugs as mentioned and loaded with DCFDA for cytosolic ROS and MitoSOX Red for mitochondrial superoxide levels. Mean fluorescence intensity was calculated for each condition. We have used one-way ANOVA with Bonferroni's multiple comparison test to compare peak to basal ratios across multiple conditions (Fig. 8*A*) and one sample or paired *t*-tests for two data set comparisons in Fig. 8.

##### Immunocytochemistry and Confocal Microscopy

Cells cultured under attached and detached conditions were fixed with 4% paraformaldehyde, and the indicated antibodies were used for staining. Confocal Z stack images were acquired at ×60 magnification in Fluoview FV10i (Olympus). Images were quantified using ImageJ by integrating pixel intensities across all *Z* sections and plotted as total intensity per cell for both attached and detached conditions. We have used one sample *t* test for statistical analysis.

##### Immunoprecipitation and in Vitro Kinase Assay

Cells grown in attached or detached conditions for the time periods mentioned were lysed with a lysis buffer containing sodium fluoride, sodium pyrophosphate, EDTA, EGTA, DTT, 1% Triton X-100, benzamidine hydrochloride, and PMSF. Sheep anti-AMPK antibody (a kind gift from Dr. Grahame D. Hardie) was used to immunoprecipitate AMPK from 120 μg of lysate. Subsequently, *in vitro* kinase reaction was carried out with AMPK substrate, AMARA, for 30 min at 37 °C. The reaction products were spotted on P81 phosphocellulose paper and washed with 1% phosphoric acid and water. Radioactive ^32^P incorporated was measured in a liquid scintillation counter. We have used one-sample Student's *t* test for statistical analysis.

##### Immunohistochemistry (IHC)

A total of 35 breast cancer and 25–30 adjacent normal tissue samples were obtained from previously untreated grade III invasive ductal carcinoma cases. The paraffin blocks were collected from Kidwai Memorial Institute of Oncology (Bangalore, KA, India). Medical Ethics Committee (Institutional Review Board of Kidwai Memorial Institute of Oncology) and Institutional Human Ethics Committee (IISc; Bangalore, KA, India) approved the study. CaMKKβ (Pierce PA530558), LKB1 (Cell Signaling Technology 3570S), and pACC (Cell Signaling Technology 11818S) antibodies were used at 1:100 dilution. IHC was performed as described previously (24). Scoring was performed by an experienced histopathologist (U. A.). Unpaired Student's *t* test was used to test for significant differences between groups. Fisher's exact test was used to test the correlation between LKB1 and pACC.

##### Statistical Analysis

All statistical analyses were performed using GraphPad Prism 5.0 software. All data are presented as means ± S.E. of the mean (S.E.). *p* values <0.05 were considered to be statistically significant. Statistical analysis was done using one-sample Student's *t* test for data representing fold change over control. For other experiments, paired or unpaired, Student's *t* test was used based on the experimental design; *** represents *p* < 0.001; ** represents *p* < 0.01; and * represents *p* < 0.05. For experiments with three groups, one-way ANOVA with Bonferroni's multiple comparison test was used. For time course experiments of ROS and calcium under different treatments, two-way repeated measures ANOVA with Bonferroni's post hoc test was used. For IHC experiments, unpaired *t* tests were used to test for significant differences between groups, and Fisher's exact test was used to test for correlation.

## Author Contributions

A. S. designed, performed, and analyzed the experiments and wrote the paper. A. R. conceived and coordinated the study and wrote the paper. U. A. helped with acquisition, analysis, and interpretation of immunohistochemistry data. All authors analyzed the results and approved the final version of the manuscript.

## Footnotes

3 The abbreviations used are:

ECM: extracellular matrix
AMPK: AMP-activated protein kinase
ROS: reactive oxygen species
CaMKK: Ca^2+^/calmodulin-dependent protein kinase kinase
LKB1: liver kinase B1
BAPTA: 1,2-bis(*o*-aminophenoxy)ethane-*N,N,N*′*,N′*-tetraacetic acid
MCI-186: 3-methyl-1-phenyl-2-pyrazolin-5-one
STO-609: 7-oxo-*7H*-benzimidazo[2,1-a]benz[de]isoquinoline-3-carboxylic acid
NAC: *N*-acetyl cysteine
DCFDA: 2′,7′-dichlorofluorescein diacetate
RGD: arginylglycylaspartic acid
ANOVA: analysis of variance
ER: endoplasmic reticulum
SOCE: store-operated calcium entry
ITPR: inositol triphosphate receptor
2-APB: 2-aminoethoxydiphenyl borate
FAK: focal adhesion kinase
IHC: immunohistochemistry
ACC: acetyl-CoA carboxylase
pACC: phospho-ACC.
